# Pancreatic Lymphoepithelial Cyst With High ¹⁸F-Fluorodeoxyglucose (FDG) Uptake Mimicking Malignancy: A Case Report and Literature Review

**DOI:** 10.7759/cureus.93172

**Published:** 2025-09-25

**Authors:** Kohei Takahashi, Sayaka Daido, Yoichiro Hijikata, Yuko Someya, Kunio Hamanaka, Ryo Kuwahara, Masashi Kuroda, Tsuyoshi Ito, Koki Moriyoshi, Seiko Kasahara

**Affiliations:** 1 Department of Diagnostic Radiology, National Hospital Organization Kyoto Medical Center, Kyoto, JPN; 2 Department of Diagnostic Pathology, National Hospital Organization Kyoto Medical Center, Kyoto, JPN

**Keywords:** 18f-fluorodeoxyglucose positron emission tomography (18f-fdg pet), cheerios-like appearance, enhanced solid components, glucose transporter 1, glut1, lec, lymphoid follicle, magnetic resonance imaging (mri), pancreatic cystic tumor, pancreatic lymphoepithelial cysts

## Abstract

We present a case of pancreatic lymphoepithelial cysts (LECs) with solid components that demonstrated ^18^F-fluorodeoxyglucose (FDG) uptake on positron emission tomography (PET). Reports of LECs with FDG uptake on PET are extremely limited, and their interpretation may be challenging due to potential confusion with malignancy. An 84-year-old man presented with nausea and abdominal discomfort. Laboratory tests revealed elevated carcinoembryonic antigen (CEA) and cancer antigen 19-9 (CA19-9) levels. Imaging studies showed a cystic mass arising from the pancreatic tail with enhancing solid components and a thickened wall. FDG PET demonstrated high uptake (maximum standardized uptake value (SUV_max_) = 8.36) in the solid portion and thickened wall. Magnetic resonance imaging (MRI) showed multiple subcentimeter nodules with heterogeneous signal intensity and restricted diffusion. Magnetic resonance cholangiopancreatography demonstrated no obvious communication with the main pancreatic duct. The patient underwent surgery due to malignancy concerns, but histology confirmed benign LEC. The solid components consisted of lymphoid follicular hyperplasia without malignant features. In our case, the uptake corresponded to areas of follicular hyperplasia. FDG is taken up via glucose transporter type 1, which is known to be expressed in follicular dendritic cells, possibly explaining the PET findings. LECs are benign, but may exhibit FDG uptake due to lymphoid tissue proliferation, mimicking malignancy. Recognizing this phenomenon, in conjunction with a comprehensive interpretation of MRI findings, may improve diagnostic accuracy.

## Introduction

Lymphoepithelial cysts (LECs) of the pancreas are rare benign lesions histologically characterized by keratinizing squamous epithelium surrounded by dense lymphoid tissue [1], accounting for approximately 0.5% of pancreatic cystic lesions [2]. They are incidentally discovered in slightly less than half of the cases [3].

Serum cancer antigen 19-9 (CA19-9) elevation is observed in approximately half of cases, often raising concerns for malignancy. Fine-needle aspiration (FNA) has been reported to be useful in the differential diagnosis [3]. LECs typically appear on imaging as well-circumscribed cystic lesions without enhanced solid components [4]. Herein, we report a rare case of pancreatic LECs presenting with 18^F^-fluorodeoxyglucose (FDG)-avid solid components in positron emission tomography (PET), which posed significant diagnostic challenges in distinguishing it from malignancy.

## Case presentation

An 84-year-old man presented with a one-week history of nausea and abdominal discomfort. Laboratory tests revealed that carcinoembryonic antigen (CEA) was 3.8 ng/mL (reference range: 0.0-3.0 ng/mL) and CA19-9 was 257 U/mL (reference range: 0.0-30.9 U/mL). Elevated tumor markers raised concern for a malignant tumor.

**Figure 1:**
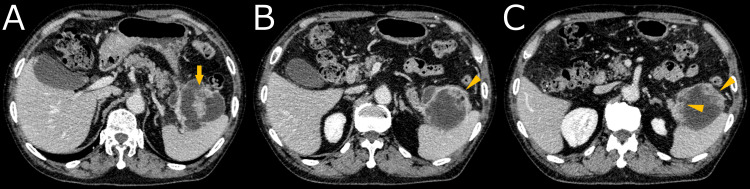
Contrast-enhanced computed tomography A cystic mass protruding from the tail of the pancreas, with enhanced solid components (arrow in A) and irregular thickening of cyst wall (arrowhead in B, C).

**Figure 2:**
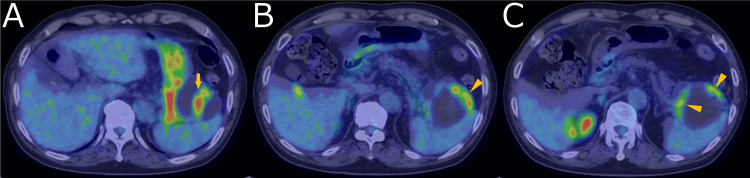
FDG-positron emission tomography Intense FDG uptake (SUVmax = 8.36) corresponding to the solid components (arrow in A) and irregular thickening of cyst wall (arrowhead in B, C), raising suspicion for malignancy. FDG: ¹⁸F-fluorodeoxyglucose

**Figure 3:**
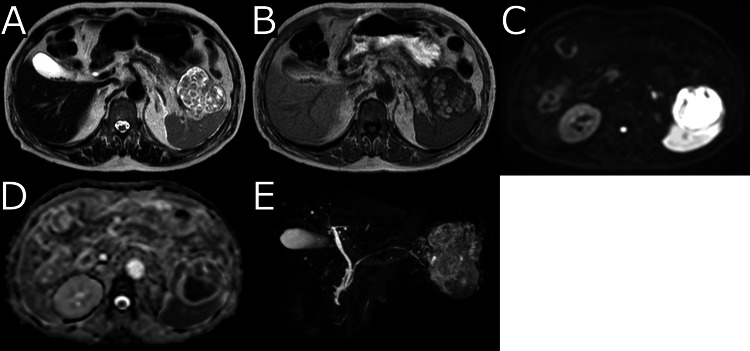
Magnetic resonance imaging Multiple subcentimeter nodules within the cyst, exhibiting heterogeneous intermediate intensity on T2-weighted image (A) and heterogeneous high intensity on T1-weighted image (B). Diffusion-weighted image demonstrated diffusion restriction (C), with an apparent diffusion coefficient value of approximately 0.400 ×10-3mm2/s (D). Magnetic resonance cholangiopancreatography demonstrated no apparent communication with the main pancreatic duct (E).

Contrast-enhanced computed tomography (CECT) demonstrated a cystic mass protruding from the tail of the pancreas, with enhanced solid components and an irregularly thickened wall (Figure 1). FDG PET revealed high FDG uptake in the solid components (SUV_max_ = 8.36) and the thickened cyst wall (Figure 2). MRI revealed multiple subcentimeter nodules within the cyst, exhibiting heterogeneous intermediate intensity on T2-weighted image (T2WI) and heterogeneous high intensity on T1-weighted image (T1WI). Diffusion-weighted image (DWI) demonstrated diffusion restriction, with an apparent diffusion coefficient (ADC) value of approximately 0.400 ×10-3mm^2^/s. Magnetic resonance cholangiopancreatography demonstrated no apparent communication with the main pancreatic duct (Figure 3).

Based on these imaging findings, the lesion was interpreted as a cystic neoplasm with solid mural nodules and intracystic hemorrhage. Although no clear communication was observed between the lesion and the main pancreatic duct, the large size of the lesion made it difficult to exclude the possibility of communication with a small branch duct. Additionally, FDG uptake in the solid component prompted consideration of intraductal papillary mucinous neoplasm (IPMN) in the differential diagnosis. The patient underwent distal pancreatectomy with splenectomy.

**Figure 4:**
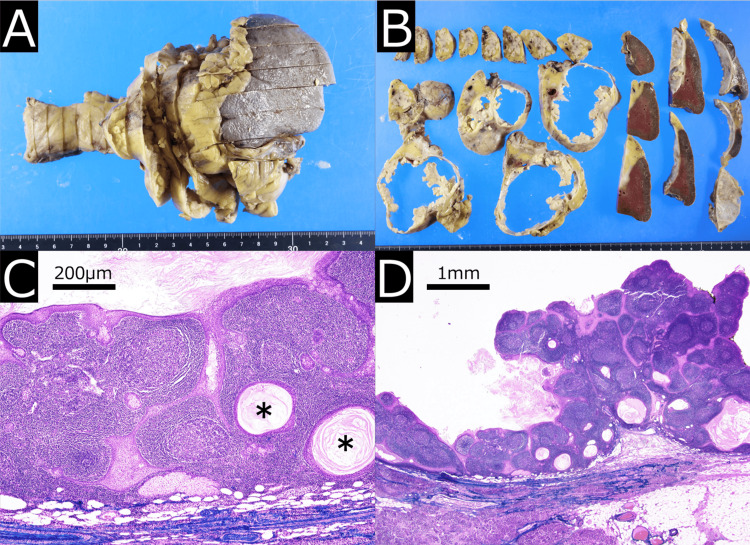
Histopathological Findings Resected specimen is seen as a cystic mass sized 10cm in diameter in the pancreatic tail. Multiple white solid nodules protrude into the cystic cavity (A, B). H&E staining shows that the cyst wall is lined by well-differentiated stratified squamous epithelium with underlying lymphoid stroma containing lymphoid follicles (C: ×100). Small cysts containing keratinous material (asterisks in C) are observed. The solid components and irregularly thickened cyst wall were composed of markedly lymphoid tissue proliferation (D: ×20). No malignant features are identified.

Gross examination revealed a cystic mass measuring 10 cm in diameter in the pancreatic tail, with a white, solid nodule protruding into the cystic cavity. The cyst contained friable yellowish material. Histologically, the cyst wall was lined by well-differentiated stratified squamous epithelium underlain by lymphoid stroma, including lymphoid follicles. Small cysts containing keratinous material were also observed. The solid components and irregularly thickened cyst wall were composed of lymphoid tissue proliferation. No malignant features were identified (Figure 4). The cyst was adherent to the surrounding organs. Keratin material was observed outside the cyst, surrounded by leukocytes, suggesting chronic inflammation due to cyst wall rupture. However, no clusters of leukocytes indicative of inflammation were observed within the cyst. Based on these findings, the final pathological diagnosis was pancreatic lymphoepithelial cysts. The postoperative course was uneventful, and the patient was discharged on postoperative day 19 without any abdominal symptoms. No recurrence has been observed over the six years following surgery.

## Discussion

Pancreatic LECs are rare benign lesions, lined with mature keratinizing squamous epithelium and surrounded by lymphoid tissue [1], accounting for approximately 0.5% of pancreatic cystic lesions [2]. To date, no cases of malignant transformation have been reported [3].

Pancreatic LECs mainly affect middle-aged to elderly men (male-to-female ratio ~3.5:1), are sometimes incidentally detected, and may show elevated CA19-9 and nonspecific MRI findings [3]. FNA may obviate unnecessary surgery in many cases [3], but in our case, tumor markers and FDG uptake suggested malignancy, making surgery unavoidable. The elevated CA19-9 is considered to result from its expression in the squamous epithelium constituting the wall of pancreatic LECs.

Imaging features of LECs may vary, with lesions appearing as unilocular or multilocular cysts, and they tend to be exophytic from the pancreatic parenchyma [4]. It is also characteristic that it is not accompanied by pancreatic duct dilation [5]. On MRI, signal intensity varies depending on the lesion's contents; however, the presence of multiple nodules smaller than 1 cm, showing central low signal and peripheral high signal on T2WI, referred to as a “cheerios-like appearance,” suggests keratinous material and is considered a finding specific to LECs [6]. In retrospective observation of this case, the heterogeneous signal intensities observed on T1WI and T2WI likely reflect the presence of keratin produced by the epithelial lining of the cyst [7].

We further explored the correlation between imaging and histopathological features of the solid components. To the best of our knowledge, five reports have described pancreatic LECs with enhanced solid components. Some reports included multiple cases, and, excluding our case, a total of nine cases have been identified in the literature [5,8-11]. In seven of these cases [5,10-11], detailed comparisons between images and histopathological findings were not provided. However, in those cases, histological examination consistently revealed stratified squamous epithelium with marked subepithelial lymphocytic infiltration, without evidence of malignancy. In the remaining two cases [8,9], a correlation between imaging and histological findings of the solid components was described. Sugiura et al. reported that the solid components exhibited a papillary structure similar to that of the cyst wall, with some interstitial connective tissue [8], as observed in our case. Maekawa et al. reported a solid component lined by squamous epithelium surrounded by a layer of lymphoid tissue, without atypical cells [9].

A multicenter pathological case series by Adsay et al., which included 12 cases of pancreatic LECs, found that the epithelium formed a complex reticular pattern that separates individual lymphoid follicles. This was prominent in some areas, forming macroscopic nodularity in the cyst lining [2]. Taken together with the histopathological correlation of our case, this suggests that the solid components observed on imaging could correspond to proliferated lymphoid follicles.

FDG PET findings in pancreatic LECs have rarely been reported. Excluding our case, nine case reports of pancreatic LECs with preoperative FDG PET imaging have been documented [9,12-19], among which four demonstrated increased FDG uptake [9,12-14] (Table 1). One of these four cases discussed the pathological correlation, attributing the FDG uptake to abscess formation rather than the LECs themselves [14]. In the present case, histopathological examination suggests chronic inflammation due to cyst wall rupture. However, no clusters of leukocytes indicative of inflammation were observed within the LECs, and thus, the increased FDG uptake in the solid components and areas of wall thickening cannot be attributed to inflammatory accumulation. In two of these four cases, no detailed discussion was provided comparing the FDG uptake sites with histopathological findings or with other imaging [12,13]. The case reported by Maekawa et al. showed FDG accumulation in a papillary-enhancing solid component, which histologically corresponded to squamous epithelium surrounded by a layer of lymphoid tissue [9], findings that are consistent with our case.

**Table 1:** Case reports of pancreatic lymphoepithelial cysts with FDG-PET FDG: ¹⁸F-fluorodeoxyglucose; PET: positron emission tomography; NA: not available; +: presence; −: absence; WNL: within normal limits; LEC: lymphoepithelial cyst; NAC: neoadjuvant chemotherapy

Author(s)	Year	Age/Sex	Location	Size(cm)	CA19-9(U/mL)	Abnormal FDG uptake (SUVmax early / delayed)	Atypical CT/MRI findings	Notable pathological findings	Surgical outcome
Maekawa et al. [9]	2009	58/M	Body	5.0	NA	+ (5.8 / NA)	Enhancing intracystic projection	Papillary projection without atypia; structure same as cyst wall	Uneventful
Yanagimoto et al. [18]	2013	53/M	Tail	4.1	WNL	−	None	None	Uneventful
Sasaki et al. [17]	2014	54/M	Neck	3.2	WNL(28.9)	−	Increased in size (2.2→3.2 cm/4 years); Enhancing septum	None	NA
Ryu et al. [19]	2015	44/F	Tail	6.4	1690	−	With internal septa	Elevated intracystic CEA level (618 ng/mL)	Uneventful
Satoh et al. [12]	2015	63/M	Body	6.0	85.5	+ (2.7 / NA)	Increased in size from 6 cm to 6.5 cm over a period of 1 year	Atypical cells on fine-needle biopsy cytology	NA
Iguchi et al. [14]	2021	65/M	Tail	3.6 and 1.5	WNL(6.1)	+ (13.1 / 17.4)	Two adjacent ring-enhancing masses (larger one newly appeared within 6 months)	LEC with chronic abscess; neutrophilic infiltration of lymphoid tissue extending from abscess to LEC	Uneventful
Murokawa et al. [16]	2022	70/M	Head	2.0	WNL(14.1)	−	None	None	Transient pancreatic leak
Sharma et al. [15]	2023	70/M	Tail	8.0	53	−	Increased in size (5.5 to 8cm, during NAC)	None	NA
Katsura et al. [13]	2024	72/M	Tail	3.0	WNL(<2.1)	+ (2.07 / 2.88)	Enhancing solid component	None	NA
Current case	2025	84/M	Tail	7.2	257	+ (8.36 / NA)	Increased in size (6.6 to 7.2 cm/2 years and 7 months); Enhancing solid component and irregular thickening of cyst wall	Marked lymphoid proliferation in solid components and thickened cyst wall	Uneventful

In the present case, FDG uptake was localized to areas of lymphoid follicular hyperplasia, both in the solid components and in the irregularly thickened cyst wall. FDG is taken up into cells via glucose transporter type 1 (GLUT1). GLUT1 is expressed in follicular dendritic cells within secondary lymphoid follicles [20].

As previously noted, few studies have examined the relationship between LEC and FDG uptake [9,12-14]. However, multiple high-quality studies show that FDG-PET is the most powerful imaging tool for distinguishing between benign and malignant lesions. Srinivasan et al. reported that FDG-avid pancreatic cystic lesions are diagnosed as malignant with a high positive predictive value, 90% in IPMN and 85% in other cystic lesions [21]. The current report of FDG uptake in LEC (a benign lesion), considered a 'false positive' in previous studies [9,12-14], provides valuable insight into existing research on FDG-PET evaluation of pancreatic cystic lesions.

Although differentiating LECs from malignant cystic neoplasms remains challenging, careful interpretation of MRI findings, such as the presence of intracystic keratinous material producing a Cheerios-like appearance and the absence of communication with the main pancreatic duct, may contribute to improved diagnostic accuracy.

## Conclusions

Although LECs are benign lesions, the presence of a solid component with FDG uptake can raise suspicion for malignancy. In the present case, FDG uptake was associated with lymphoid follicular hyperplasia, and no malignant features were observed. It is important to recognize that FDG uptake in LECs does not necessarily indicate malignancy. Careful interpretation of MRI findings may improve diagnostic accuracy.
